# Supplementary material for: Electrospray-Assisted Fabrication of Dextran–Whey Protein Isolation Microcapsules for the Encapsulation of Selenium-Enriched Peptide
Source: Foods. 2023 Feb 27;12(5):1008. doi: 10.3390/foods12051008 (PMC10000993; doi:10.3390/foods12051008)
Supplement: Supplementary file 1 [file foods-12-01008-s001.zip › foods-2112689-supplementary.pdf]

foods

MDPI

Supplementary Materials

Electrospray-Assisted Fabrication of Dextran-Whey Protein Isolation Microcapsules for the Encapsulation of Selenium-Enriched Peptide

Jiangling He [1,†], Zhenyu Wang [1,2,†], Lingfeng Wei [1], Yuanyuan Ye [1,2], Zia-ud Din [3], Jiaojiao Zhou [1], Xin Cong [1], Shuiyuan Cheng [1], Jie Cai [1,2,*]

[1] National R&D Center for Se-Rich Agricultural Products Processing, Hubei Engineering Research Center for Deep Processing of Green Se-Rich Agricultural Products, School of Modern Industry for Selenium Science and Engineering, Wuhan Polytechnic University, Wuhan 430023, China; hejiangling@whpu.edu.cn (J.H.); wzyssy10@163.com (Z.W.); wlf1060348837@163.com (L.W.); yyyuan0127@163.com (Y.Y.); jiaojiaozhou@whpu.edu.cn (J.Z.); congxinwhpu@whpu.edu.cn (X.C.); s_y_cheng@sina.com (S.C.)

[2] Key Laboratory for Deep Processing of Major Grain and Oil, Ministry of Education, Hubei Key Laboratory for Processing and Transformation of Agricultural Products, Wuhan Polytechnic University, Wuhan 430023, China

[3] Department of Food Science and Nutrition, Women University Swabi, Swabi-23430, Khyber Pakhtunkhawa, Pakistan; zia.iukco@gmail.com (Z.D.)

† These authors contributed equally to this work

* Correspondence: caijievip@whpu.edu.cn (J.C.)

Citation: He, J.; Wang, Z.; Wei, L.; Ye, Y.; Din, Z.-u.; Zhou, J.; Cong, X.; Cheng, S.; Cai, J. Electrospray-Assisted Fabrication of Dextran-Whey Protein Isolation Microcapsules for the Encapsulation of Selenium-Enriched Peptide. *Foods* **2023**, *12*, 1008. https://doi.org/10.3390/foods12051008

Academic Editor: Jayani Chandrapala

Received: 6 December 2022
Revised: 2 February 2023
Accepted: 8 February 2023
Published: 27 February 2023

Copyright: © 2023 by the authors. Submitted for possible open access publication under the terms and conditions of the Creative Commons Attribution (CC BY) license (https://creativecommons.org/licenses/by/4.0/).

1. Materials and Methods

1.1. Materials and reagents

Dextran was purchased from Adama Reagent Co., Ltd. (Shanghai, China). Whey protein isolation was purchased from Hilmar Indegrents Co., Ltd. Selenium-enriched peptide from *Cardamine violifolia* is a kind of semi purified powder, and it was provided by Enshi Se-Run Material Engineering Technology Co., Ltd. (Enshi, China). The total Se content was determined to be 1143.65 ± 64.27 μg/g. 1,1-diphenyl-2-picrylhydrazyl (DPPH) and 2,2'-azinobis (3-ethylbenzothiazoline-6-sulfonic acid ammonium salt (ABTS) were purchased from Aladdin Reagent Co., Ltd. (Shanghai, China). Anhydrous ethanol, phosphoric acid, hydrochloric acid, nitric acid, disodium hydrogen phosphate dodecahydrate, and sodium dihydrogen phosphate dihydrate were obtained from Sinopharm Chemical Reagent Co., Ltd. Alpha-amylase, pepsin, and trypsin were purchased from McLean Biochemical Technology Co., Ltd. (Shanghai, China). CCK-8 cell proliferation and cytotoxicity assay kit were obtained from Biyuntian Biotechnology Co., Ltd. (Shanghai, China). Caco-2 cell line was obtained from Procell Life Science & Technology Co., Ltd. (Wuhan, China). Dulbecco's Modified Eagle Medium (DMEM)/high glucose cell culture medium was purchased from Cytiva Ltd. (Washington, USA). All the above reagents were analytical grade or premium pure grade, and they are used as obtained.

1.2. Characterizations

Optical images were captured by a CX40 optical microscope (Sunny Group, China). Scanning electron microscope (SEM) images were collected by a SU8000 field-emission electron microscope (Hitachi, Japan) with the acceleration voltage of 15 kV. The size distributions of particles and average diameters were obtained from the image analysis of the optical images via Image J software. The identification of crystal structure changes was recorded by an Empyrean X-ray diffractometer (XRD) (Malvern Panalytical, Holland), using Cu-Kα radiation within the 2θ range of 4°-40°. Fourier-transform infrared (FTIR) curves were obtained with a Nicolet iS50 infrared spectrometer (Thermo Scientific, USA) from 400 cm^{-1} to 4000 cm^{-1} at a resolution of 2 cm^{-1}. Thermal stability was analyzed

using a Q600 thermogravimetric analyzer (TA Instruments, USA) at a heating rate of 10 ℃/min under a nitrogen atmosphere. UV-Vis spectra were measured by a UV-1000 ultraviolet-visible spectrophotometer (Aoyi Instruments, China). These structural and functional characterizations also referred to the reported works [1-2].

The total selenium content in these optimized three DX-WPI-SP microcapsules was determined by a LC-AFS6500 atomic fluorescence spectrometer (Haiguang Instrument, China), referring to the work performed by Ye et al. [3]. Specifically, 0.1 g of SP was placed in a digestion tube, and 7 mL of acid was added. After microwave digestion, 10% (w/v) of the hydrochloric acid solution was used to dilute the solution to 10 mL, and the atomic fluorescence spectrometer was applied to determine the total selenium content. A standard curve was gathered with selenium standard solutions with different concentrations.

The formula used to determine the loading rate was as follows:

$$\text{Loading rate (\%)} = (W_2/W_1) \times 100 \quad (1)$$

Where W_1 is the total mass weight of the DX-WPI-SP microcapsules, and W_2 is the mass weight of the SP loaded in the DX-WPI-SP microcapsules.

1.3. Antioxidant activity evaluation

1.3.1. DPPH radical scavenging assay

The DPPH radical scavenging ability was calculated by the following formula:

$$\text{DPPH radical scavenging rate (\%)} = (1 - (A_1 - A_2)/A_0)) \times 100 \quad (2)$$

Where A_0 is the absorbance of the control (deionized water) group; A_1 is the absorbance of the sample group mixed with DPPH; A_2 is the absorbance of the blank (sample without DPPH) group.

1.3.2. ABTS radical scavenging ability assay

The ABTS radical scavenging ability was calculated by the following formula:

$$\text{ABTS radical scavenging rate (\%)} = (1 - (A_1 - A_2)/A_0)) \times 100 \quad (3)$$

Where A_0 is absorbance value of the control group (DI water); A_1 is the absorbance of the mixture (The sample in ABTS work solution); A_2 is the absorbance value of the sample without ABTS.

1.4. Stability of DX-WPI-SP microcapsules against pH changes

Kinetic equation of release:

$$\text{Zero order: } Q = Q_0\text{-}k_0^t \quad (4)$$

$$\text{First order: } Q = Q_\infty[1\text{-}\exp(\text{-}k_1t)] \quad (5)$$

$$\text{Higuchi: } Q = k_Ht^{1/2} \quad (6)$$

$$\text{Korsmeyer-Peppas: } Q_t/Q_\infty = k_{KP}t^n \quad (7)$$

Where Q is the cumulative amount of SP released at time t, Q_0 is the initial SP amount (t = 0); Q_∞ is the final amount of SP; Q_t represents the amount of released SP at time t. n is the diffusion index of the release mechanism of SP. k_0, k_1, k_H, and k_{KP} are release rate constants for zero order, first order, Higuchi, and Korsmeyer-Peppas kinetic models, respectively.

1.5. Cell culture and vitality assay in vitro

Cell viability was calculated according to the following formula:

$$\text{Cell viability (\%)} = ((A_2 - A_0)/(A_1 - A_0)) \times 100 \quad (8)$$

Where A0 is the absorbance in the well with medium and CCK-8 solution without cells; A1 is the absorbance in the well with cells and CCK-8 solution, and without sample; A2 is the absorbance in the well with cells, CCK-8 solution, and samples.

2. **Results** and **Discussion**

2.1. Preparation of DX-WPI-SP microcapsules

The process optimization for the preparation of DX-WPI-SP microcapsules:

To study the effects of the electrospraying parameters on the preparation of DX-WPI-SP microcapsules, these parameters (raw material concentration, feeding rate, voltage, and receiving distance) were accordingly adjusted and analyzed. Based on the microscopic morphology and particle size of the microcapsules, the preparation process of dextran (DX) microcapsules was optimized by changing the concentrations of dextran (*C*, 3-15%, w/v), feeding rates (*Q*, 0.1-2.0 mL/h), voltages (*U*, 5-25 kV), and receiving distances (*H*, 5-25 cm). In Table 1, different concentrations of whey protein isolation (WPI) were introduced into the dextran microcapsules after the treatment optimization, and three representative groups of dextran-whey protein isolation (DX-WPI) microcapsules were screened out. Finally, based on the selected DX-WPI microcapsules, a certain concentration of SP was added.

Therefore, to investigate the effects of fabrication parameters on morphology of the DX-WPI-SP microcapsules via high-voltage electrostatic spray, a series of fabrication parameters were performed and optimized. Figure S1 showed the optical images and particle size distributions of DX microcapsules with the increasing concentration of dextran. Microcapsules with the higher dextran content are more sperm-like or fibrous (Figure S1e), which was attributed to the increased viscosity in the solution having the higher concentration of DX. Figure S1f showed that the average diameter of DX microcapsules gradually increased as the more introduction of DX. When C = 6% (w/v), the obtained microtopography and average diameter of DX microcapsules are appropriate, and they showed a stable yield. As shown in Figure S2, with the increase of the feeding rate for the DX solution, the particle size of the DX microcapsules gradually increases. The excessive feeding rate can promote the formation of droplets. The Taylor cone under a higher feeding rate can form larger droplets, which may make the jetting process of the droplets unstable and form microcapsules with larger particle size [4]. When Q = 1.0 mL/h, the yield of DX microcapsules is acceptable, and showing the relatively small diameter. Figure S3 revealed that the DX microcapsules collected at the U = 5 kV or 25 kV appeared fibrous appearance accompanied by larger particle size. While at 10-20 kV, the DX microcapsules were spherical, and the particle size gradually decreases at a higher voltage. Under a quite low voltage, the electrical potential difference was not enough to overcome the surface tension function of the solution, resulting in the failure to form a stable Taylor cone and the jet-flow. Under a relatively high voltage, the accumulated free electrons were enough upon the jet to make the solution split into micro-scale droplets, where the solvent in these droplets was quickly evaporated, resulting in the formation of fibrous microcapsules [5]. Thus, both too low and too high voltages are not beneficial for the formation of microcapsules. When U = 15 kV, it was occurred that relatively small diameter and relatively low energy consumption. Electrons within the different receiving distance can affect the evaporation efficiency of the solvent during the spray. Figure S4 showed that the particle size of DX microcapsules obviously decreased with the receiving distance enlarged from 5 cm to 15 cm, and then remained stable. When H = 15 cm, the microtopography and average diameter of DX microcapsules can be approbatory. Figure S5 revealed the viscosity of the electrospraying solution increased with the more addition of WPI, resulting in the increasing particle size of DX-WPI microcapsules.

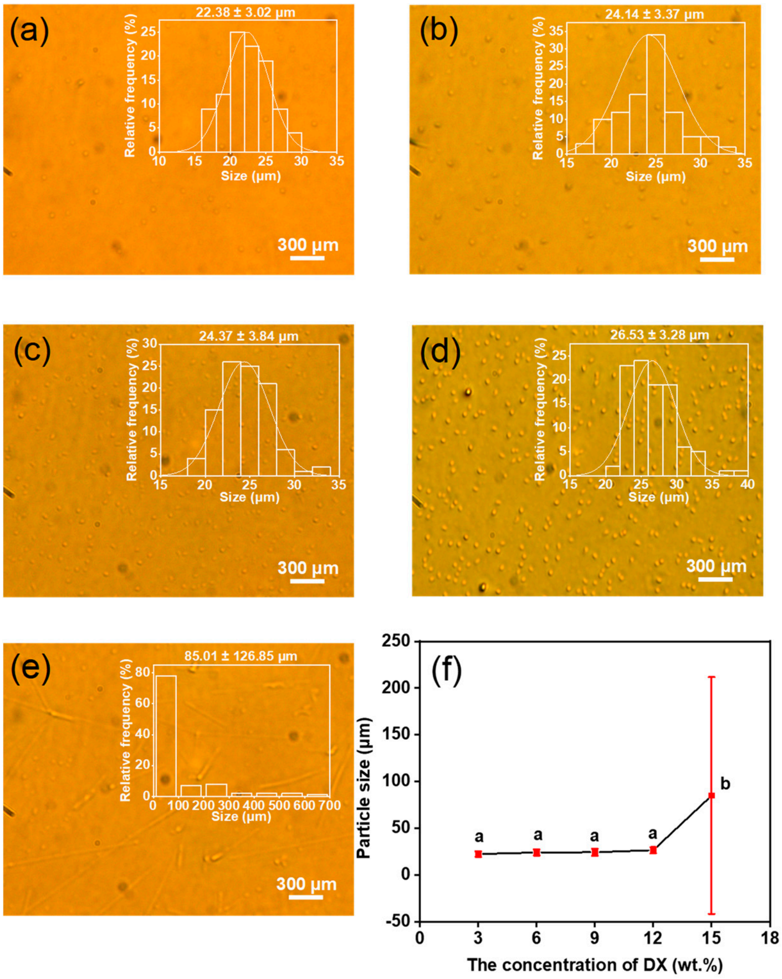

Figure S1. (a-e) Optical images and particle size distribution of dextran microcapsules with different concentrations of dextran (a-3 wt %, b-6 wt %, c-9 wt %, d-12 wt %, and e-15 wt %); (f) The average particle sizes of these dextran microcapsules with different concentrations of dextran at 3-15 wt %. Different labels represent significant differences between groups by Duncan's test ($p < 0.05$).

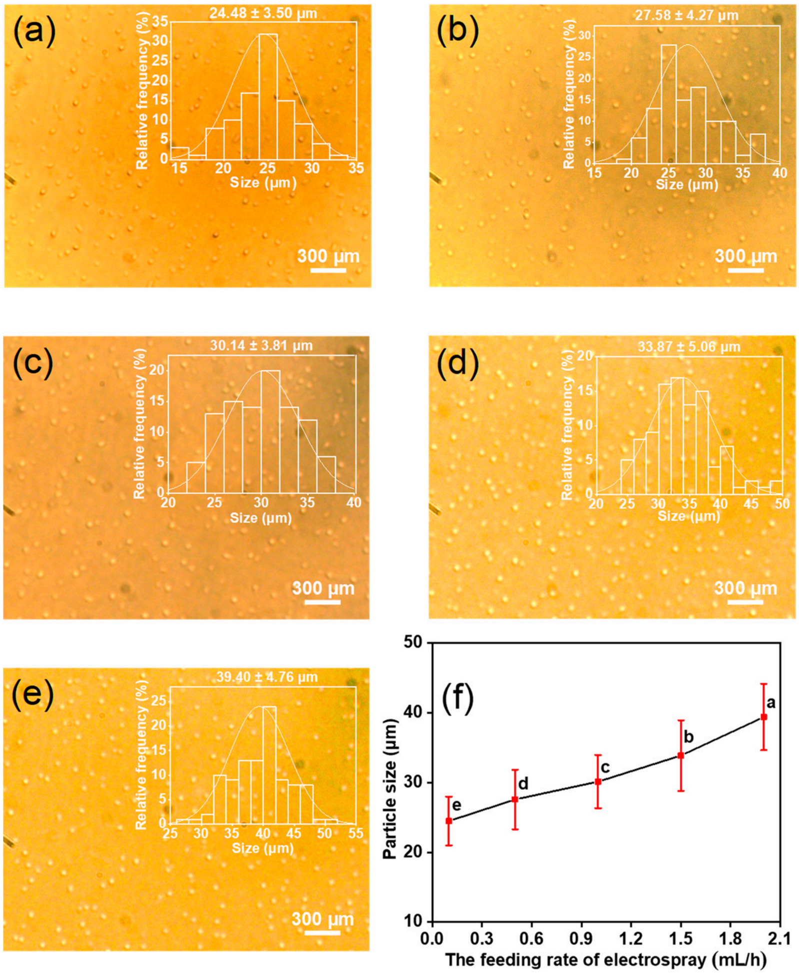

Figure S2. (a-e) Optical images and particle size distribution of dextran microcapsules with different feeding rates (a-0.1 mL/h, b-0.5 mL /h, c-1.0 mL/h, d-1.5 mL/h, and e-2.0 mL/h); (f) The average particle sizes of these dextran microcapsules with different feeding rates at 0.1-2.0 mL/h. Different labels represent significant differences between groups by Duncan's test ($p < 0.05$).

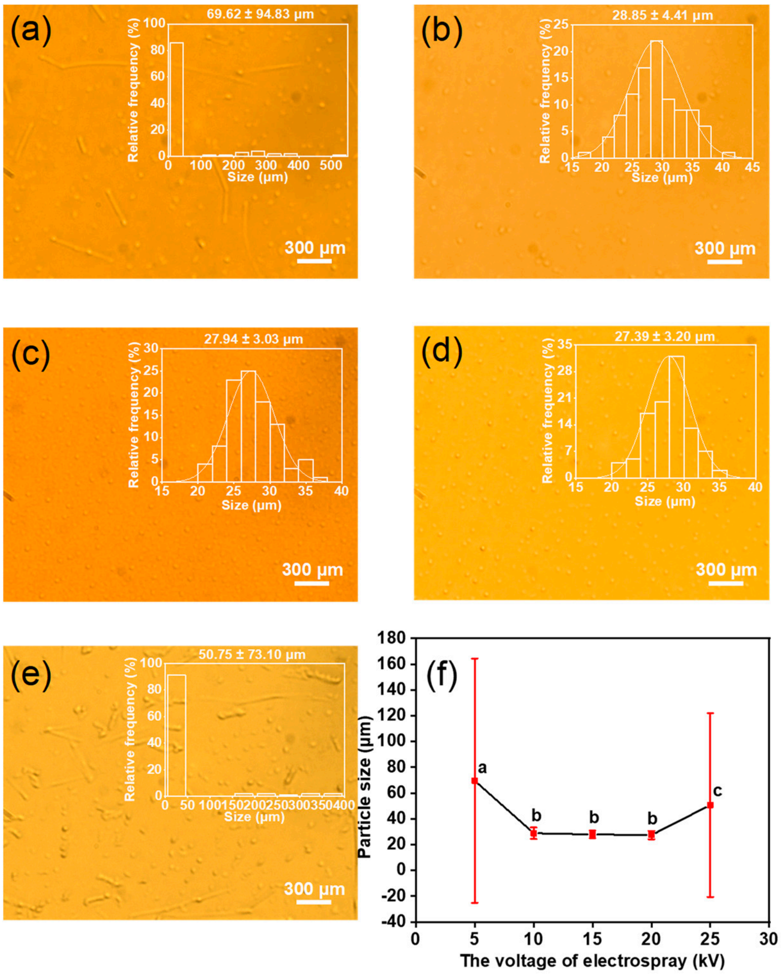

Figure S3. (a-e) Optical images and particle size distribution of dextran microcapsules with different voltage electrostatic spraying (a-5 kV, b-10 kV, c-15 kV, d-20 kV, and e-25 kV); (f) The average particle size of these dextran microcapsules with the different voltage of electrostatic spraying from 5-25 kV. Different labels represent significant differences between groups by Duncan's test ($p < 0.05$).

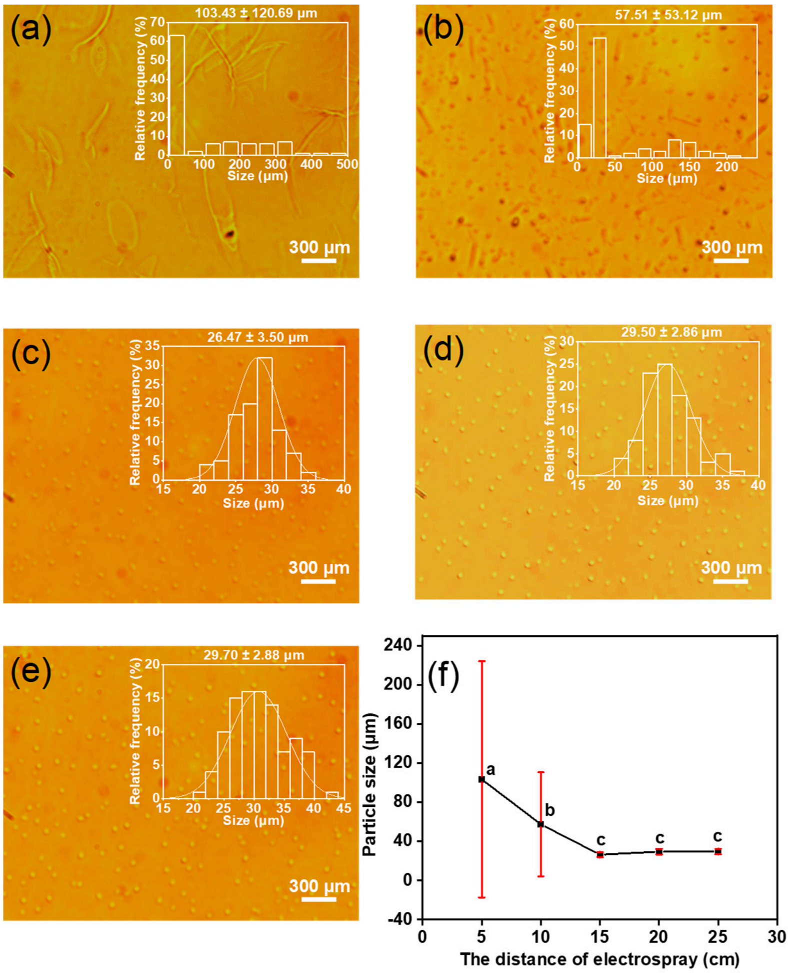

Figure S4. (a-e) Optical images and particle size distribution of dextran microcapsules with different receiving distances (a-5 cm, b-10 cm, c-15 cm, d-20 cm, and e-25 cm); (f) The average particle sizes of these dextran microcapsules with different receiving distances from 5 to 25. Different labels represent significant differences between groups by Duncan's test ($p < 0.05$).

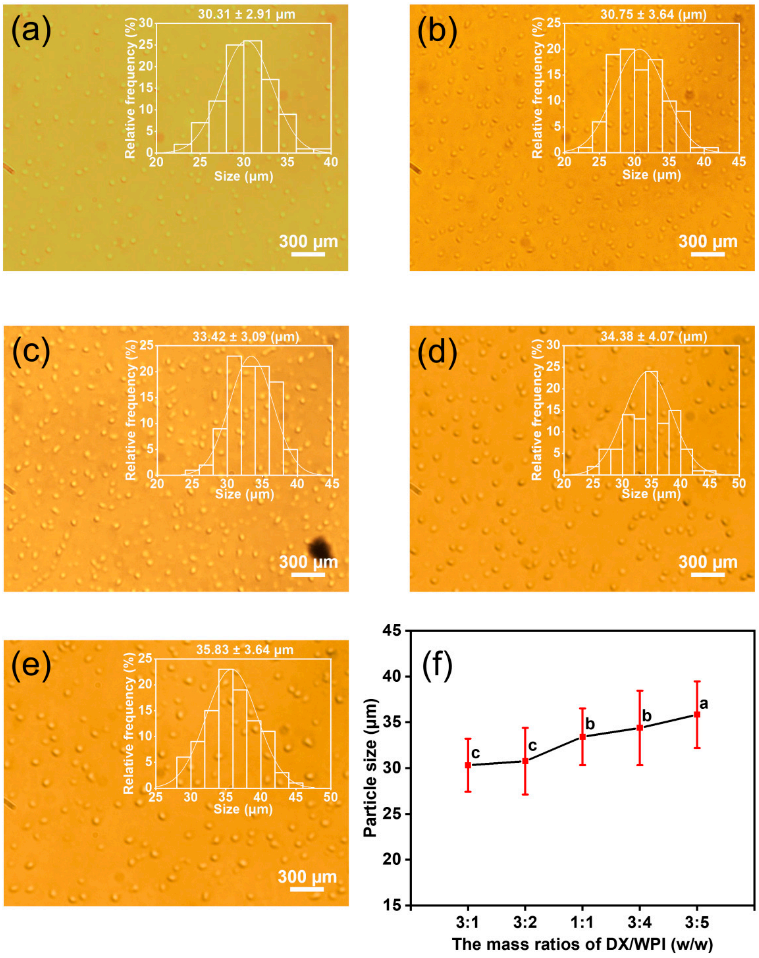

Figure S5. (a-e) Optical images and particle size distribution of DX-WPI microcapsules with different DX/WPI mass ratios (a-3:1, b-3:2, c-1:1, d-3:4, and e-3:5); (f) The average particle sizes of these DX-WPI microcapsules with different DX/WPI mass ratios from 3:1 to 3:5. Different labels represent significant differences between groups by Duncan's test ($p < 0.05$).

2.2. Controlled release properties evaluation results

Results of fitting of release kinetics:

According to the controlled release profiles recorded at various pH values, four mathematical models (zero order, first order, Higuchi, and Korsmeyer-Peppas) were applied to explain the release mechanism (Table S1) [6-8]. The correlation coefficient values of these models are shown in Table S1, where the first-level dynamic release model was more suitable to explain the release behavior of the DX-WPI-SP microcapsules under strongly acidic conditions (pH 2.0) with the determination coefficient (r^2) of 0.8611. Korsmeyer-Peppas dynamic model was more appropriate to monitor the release behavior of SP from DX-WPI-SP microcapsules under neutral and strong alkaline surroundings (pH 7.0 and pH 12.0). The certain value of the diffusion index (n) represents a typical desorption mechanism. When $0 < n < 0.45$, the diffusion model of the active substance belongs to Fick's diffusion law; when $0.45 < n < 0.89$, it is coincident with the non-Fick diffusion or abnormal release model; When $n > 0.89$, it is in accord with the skeleton dissolution mechanism [7]. As shown in Table S1, the n values (pH 7.0 and pH 12.0) were less than 0.45, which belongs to Fick's diffusion model.

Table S1. Kinetic parameters for the release process of SP from DX-WPI-SP microcapsules in different pH values.

	Zero-order	First-order	Higuchi	Korsmeyer-Peppas	
	r^2	r^2	r^2	r^2	n
pH 2.0	0.2770	0.8611	0.3827	0.5347	0.019
pH 7.0	0.4117	0.6926	0.5892	0.7451	0.032
pH 12.0	0.7419	0.6655	0.8485	0.9052	0.063

Note: r^2 represents the determination coefficient, and n is the diffusion index.

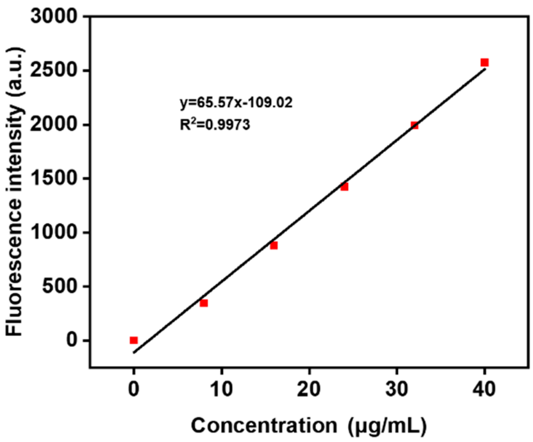

Figure S6. Standard curve for the determination of total selenium content.

References

1. Majidiyan, N,; Hadidi, M.; Azadikhah, D.; Moreno, A. Protin complex nanoparticles reinforced with industrial hemp essential oil: Characterization and application for shelf-life extension of rainbow trout fillets. *Food Chemistry: X* **2022**, 13, 100202, doi: 10.1016/j.fochx.2021.100202.
2. Hesami, S.; Safi, S.; Larijani, K.; Badi, H.N.; Abdossi, V.; Hadidi, M. Synthesis and characterization of chitosan nanoparticles loaded with greater celandine (*Chelidonium majus* L.) essential oil as an anticancer agent on MCF-7 cell line. *International Journal of Biological Macromolecules* **2022**, 194, 974-981, doi: 10.1016/j.ijbiomac.2021.11.155.
3. Ye, Y.Y.; He, J.L.; He, Z.J.; Zhang, N.; Liu, X.Q.; Zhou, J.J.; Cheng, S.Y.; Cai, J. Evaluation of the brewing characteristics, digestion profiles, and neuroprotective effects of two typical Se-enriched green teas. *Foods* **2022**, *11*, 2159, doi: 10.3390/foods11142159.
4. Anani, J.; Noby, H.; Zkria, A.; Yoshitake, T.; ElKady, M. Monothetic analysis and response surface methodology optimization of calcium alginate microcapsules characteristics. *Polymers* **2022**, *14*, 709, doi: 10.3390/polym14040709.
5. Wang, P.P.; Li, M.; Wei, D.X.; Ding, M.Z.; Tao, L.N.; Liu, X.W.; Zhang, F.P.; Tao, N.P.; Wang, X.C.; Gao, M.Y., et al. Electrosprayed soft capsules of millimeter size for specifically delivering fish oil/nutrients to the stomach and intestines. *ACS Applied & Materials Interfaces* **2020**, *12*, 6536-6545, doi: 10.1021/acsami.9b23623.
6. Garcia, P.; Vega, J.; Jimenez, P.; Santos, J.; Robert, P. Alpha-tocopherol microspheres with cross-linked and acetylated inulin and their release profile in a hydrophilic model. *European Journal of Lipid Science and Technology* **2013**, *115*, 811-819, doi: 10.1002/ejlt.201200109.
7. Ge, M.L.; Li, Y.Y.; Zhu, C.P.; Liang, G.D.; Alam, S.M.J.; Hu, G.Q.; Gui, Y.; Rashid, M.J. Preparation of organic-modified magadiite-magnetic nanocomposite particles as an effective nanohybrid drug carrier material for cancer treatment and its properties of sustained release mechanism by Korsmeyer-Peppas kinetic model. *Journal of Materials Science* **2021**, *56*, 14270-14286, doi: 10.1007/s10853-021-06181-w.
8. Wu, I.Y.; Bala, S.; Skalko-Basnet, N.; di Cagno, M.P. Interpreting non-linear drug diffusion data: Utilizing Korsmeyer-Peppas model to study drug release from liposomes. *European Journal of Pharmaceutical Sciences* **2019**, *138*, 105026, doi: 10.1016/j.ejps.2019.105026.
